# An attempt to induce an immunomodulatory effect in rowers with spirulina extract

**DOI:** 10.1186/s12970-018-0213-3

**Published:** 2018-02-20

**Authors:** Artur Juszkiewicz, Piotr Basta, Elżbieta Petriczko, Bogusław Machaliński, Jerzy Trzeciak, Karolina Łuczkowska, Anna Skarpańska-Stejnborn

**Affiliations:** 1 Department of Morphological and Health Sciences, Faculty of Physical Culture in Gorzów Wlkp. Poland, 13 Estkowskiego Str.66 – 400, Gorzów Wlkp, Poland; 2 Department of Water Sports, Faculty of Physical Culture in Gorzów Wlkp. Poland, 13 Estkowskiego Str, 66 – 400 Gorzów Wlkp, Poland; 3 Department of Pediatrics, Endocrinology, Diabetology, Metabolic Disorders and Cardiology of Developmental Age, Pomeranian Medical University, 1 Unii Lubelskiej Str, 71-252 Szczecin, Poland; 4 Department of General Pathology, Pomeranian Medical University, 72 Al. Powstanców Wlkp. Str, 70-111 Szczecin, Poland; 5 Department of Morphological and Health Sciences, Faculty of Physical Culture in Gorzów Wlkp. Poland, 13 Estkowskiego Str, 66 – 400 Gorzów Wlkp, Poland

**Keywords:** Spirulina, Supplementation, Flow cytometry, Inflammation, Strenuous exercise, Rowers

## Abstract

**Background:**

The aim of this study was to analyze the response of selected components of the immune system in rowers to maximal physical exercise, and to verify if this response can be modulated by supplementation with spirulina (cyanobacterium *Spirulina platensis*)*.*

**Method:**

The double-blind study included 19 members of the Polish Rowing Team. The subjects were randomly assigned to the supplemented group (*n* = 10), receiving 1500 mg of spirulina extract for 6 weeks, or to the placebo group (*n* = 9). The participants performed a 2000-m test on a rowing ergometer at the beginning (1st examination) and at the end of the supplementation period (2nd examination). Blood samples were obtained from the antecubital vein prior to each exercise test, 1 min after completing the test, and after a 24-h recovery period. Subpopulations of T regulatory lymphocytes (Tregs) [CD4+/CD25+/CD127-], cytotoxic lymphocytes (CTLs) [CD8+/TCRαβ+], natural killer (NK) cells [CD3-/CD16+/CD56+] and TCRδγ-positive (Tδγ) cells were determined by means of flow cytometry.

**Results:**

On the 2nd examination, athletes from the supplemented group showed neither a post-exercise increase in Treg count nor a post-recovery decrease in Tδγ cell count (both observed in the placebo group), and presented with significantly lower values of Treg/CTL prior to and after the exercise. During the same examination, rowers from the placebo group showed a significant post-recovery increase in Treg/(NK + Tδγ + CTL) ratio, which was absent in the supplemented group.

**Conclusion:**

The results of this study imply that supplementation with spirulina extract may protect athletes against a deficit in immune function (especially, anti-infectious function) associated with strenuous exercise, and may cause a beneficial shift in “overtraining threshold” preventing a radical deterioration of immunity.

## Background

Previous studies demonstrated that influence of physical exercise on the immune system may vary considerably depending on the type of training load. Regular, moderate physical exercise improves immunity, exerting a beneficial effect on the immune system [1]. However, an opposite effect may be exerted by strenuous exercise which was shown to activate immunological and endocrine mechanisms similar to those prevailing in chronic stress, trauma and sepsis [2, 3]. This may lead, in particular in high-performance athletes, to impairment of immune response and resultant greater susceptibility to infections, e.g. respiratory tract infections [4, 5]. Previous studies demonstrated that a decrease in salivary IgA concentrations and enhanced synthesis of antigen-stimulated IL-10 correlate strongly with an increase in the incidence of upper respiratory tract infections in athletes. However, the exact etiological mechanism of the immunity impairment is still not fully understood [6, 7]. Moreover, no other markers of the exercise-induced immunity impairment predisposing to upper respiratory tract infections have been established thus far [7].

Krüger et al. [8] and Adams et al. [9] showed that strenuous exercise leads to a transient increase in circulating lymphocyte count, followed by a persistent decrease in this parameter, even to the point of lymphopenia. According to these authors, after strenuous exercise, lymphocytes may migrate to some critical regions, including muscles, particularly resulting in a shift in lymphocyte subpopulations [10]. Effector lymphocytes, such as natural killer (NK) cells, cytotoxic T CD8+ cells (CTLs), TCRδγ-positive (Tδγ) cells and regulatory T cells (Tregs), are a key component of human immune system and play a crucial role in cell-mediated immune response. Interactions between cytotoxic lymphocytes and Tregs are of particular interest to researchers dealing with various pathological conditions (e.g. autoimmune diseases and neoplasms) [11–19]. In contrast, only few previous studies have addressed the problem associated with a shift in lymphocyte subpopulations and interactions between various types of immune cells in athletes. Furthermore, the results of these sparse studies are generally inconclusive.

Many recent studies dealt with Tregs, a lymphocyte subpopulation that can be identified on flow cytometry based on the expression of CD4, CD25 and FOXP3 antigens (or lack of CD 127) [20]. This growing research interest is associated with the leading role of Tregs in immune tolerance, as well as with their involvement in the control of an overactivated immune system. Under physiological conditions, Tregs represent no more than 5–10% of CD4+ lymphocytes present in peripheral blood [20, 21] . Tregs prevent autoimmune reactions, allograft rejection, food intolerance and rejection of the fetus by the maternal immune system [20]. However, aside from these beneficial effects, they may also interfere with effector function and proliferation of cells involved in the specific and non-specific immune response, such as CTLs, NK cells, monocytes, macrophages and dendritic cells [22, 23]. Available evidence suggests that overactivity of Tregs may result in immune impairment, potentially predisposing individuals to chronic infections or facilitate formation of metastases in cancer patients [13, 15, 18, 19, 24, 25]. Kunzmann et al. [26] showed that Tregs can inhibit phosphoantigen-induced proliferation of non-conventional highly-cytotoxic Tδγ cells. Furthermore, few previous studies demonstrated that repeated strenuous exercise may persistently alter absolute and relative size of lymphocyte subpopulations, causing a shift toward Tregs. This phenomenon may be inter alia reflected by an increase in the level of IL-10 synthesized by these cells, and is at least in part responsible for the resulting immunosuppression [27]. Furthermore, strenuous physical exercise may cause a decrease in the number of NK cells and CTLs [10, 28, 29].

However, the results of previous studies analyzing immunomodulatory effects of physical exercise, either in humans or in animal models, are inconclusive. Wang et al. [30] demonstrated that 6 weeks of exhaustive high-intensity exercise resulted in an increase in the number of Tregs, and altered proportions of CD4+ and CD25+ cells in murine spleen. Similar effects, however, were not observed in mice subjected to moderate-intensity exercise. These findings were partially confirmed by Wilson et al. [31] who showed that acute, intense swimming exercise contributed to an increase in the number of circulating Tregs in adolescent athletes. However, quite contradictive results were obtained by Perry et al. [32] when analyzing the effects of strenuous endurance exercise in marathon runners and half-ironman triathletes, reporting a substantial post-exercise decrease in the number of circulating Tregs.

Addition of supplements with established immunomodulatory properties to athletes’ diet may constitute a form of mild and safe intervention to restore immune balance. A cyanobacterium, *Spirulina platensis* (SPR) is a supplement with a good safety profile and established immunomodulatory potential [33, 34]. Many previous studies document beneficial effects of SPR on the immune system in both animals and humans [34, 35]. SPR is rich in protein, vitamins, minerals and unsaturated fatty acids, especially gamma-linolenic acid. Moreover, it contains phycocyanins and lipopolysaccharides, key components of cell walls in Gram-negative bacteria [36]. Many authors showed that SPR may stimulate macrophages to synthesize IL-12, a cytokine that promotes production of INT-γ by NK cells [37]. Published evidence suggests that supplementation with SPR may also prevent replication of viruses, and thus may be helpful in control of many viral infections, such as chickenpox, cytomegaly, measles, mumps, influenza and HIV [38, 39]. Løbner et al. [40] showed that supplementation with a polysaccharide extract of SPR stimulated production of IL-2, INT-γ and TNF-alpha, while down-regulating the synthesis of IL-4 in antigen-activated lymphocytes from healthy volunteers. A decrease in IL-4 production was also observed in SPR-supplemented (2 g daily for 12 weeks) patients with allergic rhinitis. Since IL-4 was previously shown to inhibit spontaneous apoptosis of Tregs, its down-regulation likely enhances programmed death of these cells. Theoretically, the resultant decrease in Treg count should promote a cell-mediated immune response [41, 42]. Indeed, a 7-day supplementation with SPR extract increased NK cell activity in healthy persons [43]. Moreover, administration of SPR enhanced antitumor activity in syngeneic tumor-implanted mice [35].

Many previous studies demonstrated that SPR may cause a shift in Th1/Th2 balance toward Th1-dominant immunity. This justifies the analysis of SPR’s effects in athletes, in whom strenuous physical exercise may impair immunity and disrupt balance between cytotoxic lymphocytes and Tregs. However, to the best of our knowledge, none of the previous studies analyzed the effects of SPR on the number of Tregs, NK cells, CTLs and Tδγ cells in this group. Nevertheless, the studies analyzing suppressive effects of Tregs and cytotoxicity of NK cells, Tδγ cells and CTLs may produce flawed results unless other components of the immune system are considered. Exposure to an extreme stressor, such as maximal exercise, results in an increase/decrease in the number of cytotoxic lymphocytes, counterbalanced with a decrease/increase in Treg count. It is the outcome of these processes, which eventually reflects the influence of exercise on immune function. Since previous studies documented a plethora of interactions (either direct or cytokine-mediated) between various lymphocyte subpopulations, we searched for possibly most accurate marker of these complex relationships. Other authors [12, 17, 26, 44] demonstrated that Treg to effector T cell ratio reflects suppressive effects of the former on various effector cells more accurately than a simple determination of their counts. To the best of our knowledge, none of the previous studies examined exercise- induced changes in these parameters in sportspersons. Therefore, the aim of this study was to analyze changes in Treg/Tδγ, Treg/NK, Treg/CTL and Treg/(NK + Tδγ + CTL) ratios in SPR-supplemented athletes subjected to strenuous exercise; to the best of our knowledge, none of the previous studies examined exercise-induced changes in these parameters in sportspersons.

## Methods

### Study population

The study included 19 men, all members of the Polish Rowing Team (15 heavy-weight and 4 light-weight rowers). Basic characteristics and sport classes of the athletes are presented in Table 1. The study was conducted between March and May, during a 6-week training camp, scheduled between the preparatory and competitive phase of the yearly training cycle. The characteristics of the training profile, such as its intensity, volume (in minutes) and type (specific, i.e. rowing: endurance, technical, speed, etc., and nonspecific: jogging, strength) were recorded on a daily basis. The intensity of the training was classified based on the lactate acid (LA) threshold (4 mmol/L), as extensive (below the LA threshold), highly intensive (above the LA threshold), and extremely intensive (control tests) (Table 2).

**Table 1:** Basic characteristics of the study groups

Parameters	Supplemented group (*n* = 10)	Placebo group (*n* = 9)
Age (years)	20.4 ± 0.84	20.0 ± 0.71
Body mass (kg)	84.4 ± 8.43	87.1 ± 5.80
Body height (cm)	192.3 ± 4.32	190.8 ± 3.89
Duration of training (years)	7.3 ± 1.3	6.8 ± 1.8

Values represent means ± standard deviations. No statistically significant differences were found for all intergroup comparisons (*P* > 0.05)

**Table 2:** Training schedule for the week preceding blood sampling during the 1st and the 2nd examination

	*Days before the 1st examination*						
	1	2	3	4	5	6	7
Total training time, min/day	120	150	200	190	210	140	120
Time rowed, min/day	110	100	100	100	70	90	100
Distance rowed, km/day	22	20	20	20	16	18	20
Training for force development, min/day	–	–	90	–	70	–	–
Extensive endurance rowing training time, min/day	70	100	100	60	40	90	100
High intensity endurance rowing training time, min/day	40	–	–	40	30	–	–
Unspecific training (running, etc.), min/day	10	50	10	90	70	50	20
	*Days before the 2nd examination*						
	1	2	3	4	5	6	7
Total training time, min/day	110	120	100	160	200	120	130
Time rowed, min/day	100	100	90	90	120	100	120
Distance rowed, km/day	20	18	18	18	20	20	20
Training for force development, min/day	–	–	–	60	60	–	–
Extensive endurance rowing training time, min/day	80	70	90	90	94	80	125
High intensity endurance rowing training time, min/day	6	9	–	–	12	20	5
Very high intensity endurance rowing training time, min/day	14	21	–	–	14	–	–
Unspecific training (running, etc.), min/day	10	20	20	10	20	20	10

### Food intake

Throughout the study period, the athletes accommodated at one of the Olympic Training Centers, whereby they had all their meals. Their regular menu consisted of a mixed diet, providing the recommended dietary allowance (RDA) of carbohydrates, proteins, fats and micronutrients (vitamins and minerals), in line with the Polish Nutrition Society guidelines [45]. Daily intakes of food, calories, fruits and vegetables were the same throughout the study period. All athletes were provided with a bottled mineral water and instructed about the necessity of immediate fluid repletion, in particular prior to and after the exercise.

The study subjects declared that they had ceased all drugs, medications and dietary supplements at least 2 weeks prior to the study, and did not use them throughout the entire study period.

### Experimental procedure

Athletes who were randomized to the supplemented group (*n* = 10) received capsules with *Spirulina platensis* extract, manufactured by GAL (Poznan, Poland)*.* A single 596-mg capsule was made of 500 mg *Spirulina platensis* containing 5.9 mg chlorophyll, 0.093 mg vitamin B_6,_ 6.5 μg vitamin K, 0.8 μg vitamin B_12_, 3.5 μg selenium and 9.0 μg iodine, coated with 96 mg gelatin. The subjects were asked to take one capsule before each of their three main meals during the day, for a period of 6 weeks, which corresponded to 1500 mg of spirulina extract per day. Athletes randomized to the placebo group (*n* = 9) received visually identical capsules with calcium gluconate (500 mg per capsule)*.*

### Training program

Training volumes (expressed in minutes per day) during a week preceding the 1st and the 2nd examination, particularly for extensive rowing, intensive rowing, kilometers, and extensive non-specific training, are shown in Table 2. During the load training phase (before the 1st examination), the training volume amounted to 1130 min· wk.^− 1^, including approximately 49.6% of extensive rowing, 26.5% of non-specific training (e.g. power training) and 23.9% of intensive rowing. Total training volume before the 2nd examination was 940 min·wk.^− 1^, and included approximately 66.9% of extensive rowing, 10.7% of intensive rowing (with 5.2% of maximum-intensity control tests) and 11.7% of land training.

### Rowing performance test

The athletes performed a controlled 2000-m time trial on the first day (prior to the supplementation) and at the end of training camp (after the supplementation). Each subject had to cover the 2000-m distance on a rowing ergometer (Concept II, Morrisville, VT USA) in as short time as possible. Because the results of both tests were taken into consideration during selection to the championship team, the athletes were well motivated to perform both tests at maximal effort. Prior to each test, the subjects performed a 5-min individual warm-up on the rowing ergometer Concept II, according to their own freely selected program.

### Sample treatment

Blood samples from the antecubital vein were collected to tubes with dipotassium ethylene diamine tetra-acetic acid (K_2_EDTA) as an anticoagulant. Blood was collected before each 2000-m test (after 7–8 h of overnight fasting), 1 min after completing the test, and after a 24-h recovery period. The samples were centrifuged at 2200 rpm for 10 min. After removing the plasma and adding 1× Lysing Buffer (BD Biosciences), the samples were incubated in darkness for 15 min. Then, a PBS buffer was added, and the cells were washed twice to remove all erythrocytes.

Moreover, capillary blood samples from an ear lobe were collected prior to and after each exercise test, to assess lactic acid (LA) level.

### Measurements

Cytometric analysis of lymphocyte subpopulations: Tregs [CD4+/CD25+/CD127-], CTLs [CD8+/TCRαβ+], NK cells [CD3-/CD16+/CD56+] Tδγ cells was conducted after their labeling with fluorochrome-conjugated antibodies from BD Biosciences. Cells obtained after hypotonic lysis of peripheral blood were incubated in darkness at room temperature for 20 min with the respective antibody at a concentration specified by the manufacturer to identify each lymphocyte subpopulation (Table 3). Then, the cells were washed twice with PBS buffer and left in darkness in 3.7% formaldehyde solution for 10 min. Afterwards, the cells were again washed with PBP buffer and 100 μL of DAPI solution (1 mg/mL, Thermo Fisher Scientific) were added to stain cell nuclei. The cells were incubated in darkness at room temperature for 5 min, washed twice, and suspended in 250 μL of PBS buffer. After labeling, the cells were analyzed with a LSRII flow cytometer from BD Biosciences, coupled with BD FACSDiva software.

**Table 3:** Antibodies used for identification of lymphocyte subpopulations

Lymphocyte subpopulation	Antibody
Regulatory T lymphocytes	FITC Mouse Anti-Human CD4
	PE Mouse Anti-Human CD25
	Alexa Fluor 647 Mouse anti-Human CD127
Cytotoxic lymphocytes	APC Mouse Anti-Human CD8
	FITC Mouse Anti-Human TCR αβ
NK cells	FITC Mouse Anti-Human CD3
	PE Mouse Anti-Human CD16
	APC Mouse Anti-Human CD56
TCRδ/γ lymphocytes	FITC Mouse Anti-Human TCRδ/γ

### Statistical analysis

Statistical analyses were performed with STATISTICA v. 10.0 software package (StatSoft, Cracow, Poland). All parameters were compared using 2 (supplementation: supplemented vs. placebo) × 3 (exercise: pre-exercise vs. post-exercise vs. post-recovery) repeated measures analysis of variance (ANOVA). Normal distribution of the study variables was verified with Shapiro-Wilk test. Whenever the result of ANOVA was statistically significant, Fisher’s *post-hoc* test was conducted to identify the source of significant differences. Anthropometric characteristics of the study groups were compared with Student’s unpaired *t*-test. Except from the rowing time, the results of the 2000-m tests performed prior to and after the supplementation were compared with Student’s paired *t*-test, and intergroup comparisons were conducted with Student’s unpaired *t*-test. The results of the 2000-m simulated rowing test were subjected to one-way ANOVA. Statistical characteristics of the study variables are presented as means ± SD, and the threshold of statistical significance for all tests was set at *P* < 0.05.

## Results

Athletes from the supplemented group and the placebo group did not differ significantly in terms of their mean age, height, body weight, and years of training (Table 1).

No significant intergroup differences were found in mean power output and total run time during the 2000-m test performed at the beginning of the training camp. Furthermore, no significant differences in the pre- and post-test blood LA levels were documented when the results of the 1st examination were compared to those of the 2nd examination (Table 4).

**Table 4:** Changes in 2,000 m rowing ergometer performance before and after supplementation

Parameters	Supplemented group (*n*=10)		Placebo group (*n*= 9)	
	Before	After	Before	After
Power (Watt) (W x kg^-1^)	425 ± 30.89 5.05 ± 0.35	425 ± 28.98 5.09 ± 0.29	409 ± 26.8 4.71 ± 0.41	414 ± 34.24 5.10 ± 0.27
LA_min_ (mmol x L^-1^)^a^	1.8 ± 0.23	1.9 ± 0.27	1.7 ± 0.23	1.7 ± 0.34
LA_max_ (mmol x L^-1^)^a^	13.8 ± 1.96	10.7 ± 1.87	13.6 ± 2.11	10.2 ± 3.34
Time (s)	375.2 ± 9.43	374.4 ± 8.63	379.6 ± 8.35	378.1 ± 10.45

Values represent means ± standard deviations. ^a^LA, lactate acid. No statistically significant differences were found between the pre- and post-supplementation results (*P*<0.05)

**Fig. 1:**
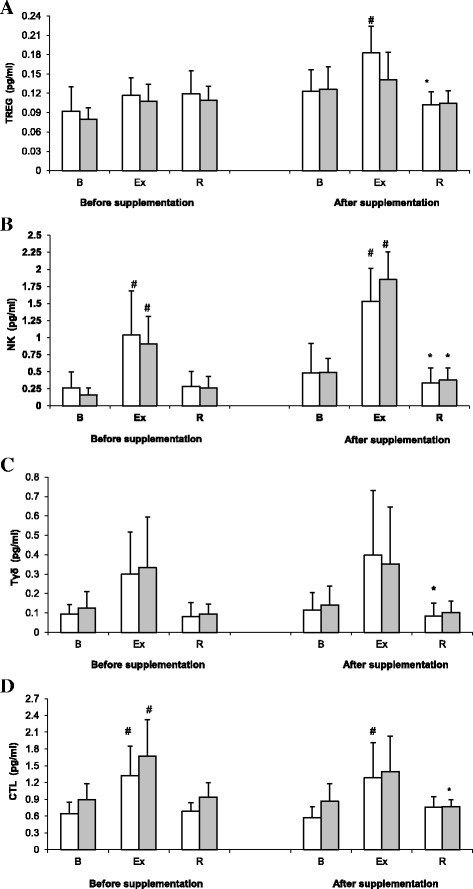
Changes in Treg (**a**), NK cell (**b**), Tδγ cell (**c**) and CTL (**d**) counts during exercise tests performed prior to and after the supplementation (mean ± *SD*). *Note*. Tregs = regulatory T cells; NK = natural killer cells; Tδγ *= gamma delta T cells*; CLT = cytotoxic T lymphocytes; gray box - SUPPL = supplemented group; □ - PLA = placebo group; B = baseline; Ex = post-exercise; R = after a 1-day recovery; # - significantly different compared to the baseline level; * - significantly different compared to the post-exercise level

Treg counts determined during the 1st and the 2nd examination are shown on Fig. 1a. ANOVA revealed a significant main effect of exercise on Treg count (*p* < 0.001). During the 2nd examination, athletes from the placebo group showed a significant post-exercise increase in Treg count, with subsequent normalization of this parameter post-recovery (*p <* 0.05). In contrast, no significant exercise-induced changes in Treg count were observed in the supplemented group during the 2nd examination, as well as in both study groups during the 1st examination. Based on the results of ANOVA, spirulina supplementation did not exert a statistically significant effect on Treg count (main effect, *p* = 0.069).

NK cell counts determined prior to and after supplementation with SPR extract are shown in Fig. 1b. ANOVA demonstrated a significant main effect of exercise on NK cell count (*p* < 0.001). During both the 1st and the 2nd examination, a significant post-exercise increase in this parameter was observed in both the supplemented group and the placebo group, followed by a post-recovery normalization (solely during the 2nd examination).

Tδγ cell counts in the study subjects are presented on Fig. 1c. Like for other previously mentioned lymphocyte subpopulations, ANOVA demonstrated a significant main effect of exercise on Tδγ cell count (*p* < 0.001). During the 1st examination, pre-exercise, post-exercise and post-recovery Tδγ cell counts did not differ significantly, in the supplemented group or placebo group. However, during the 2nd examination, a significant post-exercise decrease in Tδγ cell count was observed in the placebo group. Nevertheless, the main effect of supplementation on Tδγ cell count was not statistically significant (*p* = 0.691).

ANOVA demonstrated that exercise exerted a significant main effect on CTL count (*p* < 0.001). Irrespective of the group, a significant post-exercise increase in CTL count was observed during the 1st examination (Fig. 1d). However, during the 2nd examination, the significant post-exercise increase in this parameter was observed in the placebo group, but not in the supplemented group. Furthermore, athletes from the supplemented group showed a post-recovery decrease in CTL count during the 2nd examination. However, the main effect of supplementation on CTL count did not turn out to be statistically significant on ANOVA (*p* = 0.333).

**Fig. 2:**
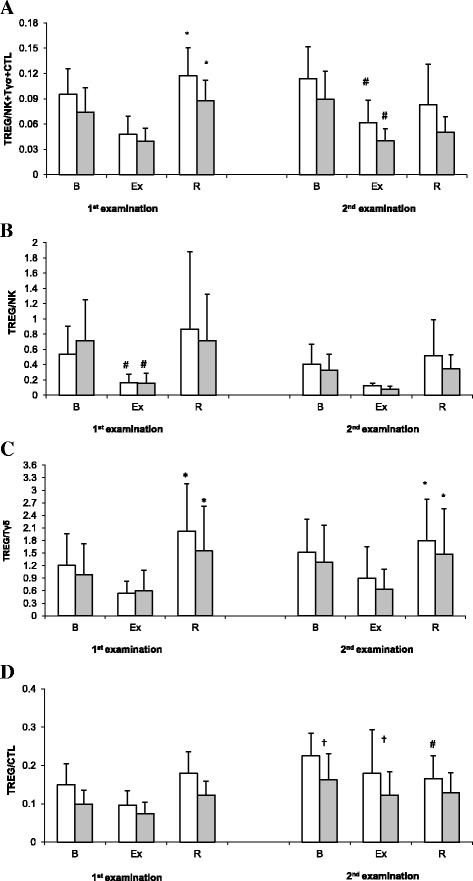
Treg/(NK + Tδγ + CTL) ratio (**a**), Treg/NK ratio (**b**), Treg/Tδγ ratio (**c**) and Treg/CTL (**d**) ratio during exercise tests performed prior to and after the supplementation (mean ± *SD*). *Note*. Tregs = regulatory T cells; NK = natural killer cells; Tδγ *= gamma delta T cells*; CTL = cytotoxic T lymphocytes; gray box - SUPPL = supplemented group; □ − PLA = placebo group; B = baseline; Ex = post-exercise; R = after a 1-day recovery; # - significantly different compared to baseline level; * - significantly different compared to post-exercise level; † − significantly different compared to PLA group

Values of the Treg/(NK + Tδγ + CTL) ratio are presented on Fig. 2a. During the 1st examination, a significant post-recovery increase in Treg/(NK+ Tδγ + CTL) ratio was observed in both study groups. In turn, during the 2nd examination, post-exercise values of Treg/(NK+ Tδγ + CTL) ratio in the supplemented group and the placebo group turned out to be significantly lower than pre-exercise values of this parameter. Nevertheless, ANOVA showed that the main effect of supplementation on Treg/(NK+ Tδγ + CTL) ratio was not statistically significant (*p* = 0.563).

Values of Treg/NK ratio were modulated solely by exercise. Specifically, the post-exercise values of this parameter in both study groups were significantly lower than pre-exercise levels (Fig. 2b). However, this relationship was observed solely during the 1st examination, as no significant exercise-induced changes in Treg/NK ratio were found during the 2nd examination. Supplementation exerted no significant effect on the values of Treg/NK ratio.

Exercise-induced changes in Treg/Tδγ ratio are presented in Fig. 2c. Irrespective of the study group, strenuous physical exercise contributed to statistically significant changes in this parameter during both the 1st and the 2nd examination (main effect, *p* < 0.001). The post-recovery increase in Treg/Tδγ ratio in the supplemented and control group corresponded to 154% and 274%, respectively, prior to the supplementation, and to 133% and 101%, respectively, after the supplementation. Based on the results of ANOVA, spirulina supplementation did not exert a statistically significant effect on Treg/Tδγ ratio (main effect, *p* = 0.129).

Statistical analysis showed that both exercise and supplementation exerted a significant main effect on the values of Treg/CTL ratio (*p* = 0.01 and *p* < 0.001, respectively) (Fig. 2d). Irrespective of the group, no statistically significant changes in this parameter were documented during the 1st examination. However, during the 2nd examination, a significant post-recovery decrease in this parameter was observed in the supplemented group, but not in the placebo group. Furthermore, post-exercise and post-recovery values of Treg/CTL ratio in supplemented athletes turned out to be significantly lower than in subjects from the placebo group.

## Discussion

During the 2nd examination, a significant post-exercise increase in Treg count was observed solely in the placebo group, with subsequent normalization of this parameter after a 24-h recovery (Fig. 1a). Since lymphocytes are known to constantly migrate between the blood and other tissues, the post-exercise increase in circulating Tregs observed in the placebo group likely reflected a concomitant increase in the tissue count of these cells (Table 2). Previous research showed that Tregs may leave the circulation and migrate to lymph nodes and inflamed tissues [46, 47], whereby they mitigate the activity of cytotoxic cells and antigen presentation by dendritic cells (DCs) [48]. This local suppressive effect of Tregs may exert an unfavorable effect on systemic immunity. Morgado et al. [49] demonstrated that an increase in the training loads of swimmers contributed to a significant reduction of cytokine synthesis by monocytes and dendritic cells. Immune impairment associated with heavy training loads may occur primarily in immunologically active tissues, at least partially explaining the difficulties in identification of accurate markers of immunosuppression that could be determined in peripheral blood. Published evidence suggests that the immune impairment caused by escalation of training loads has no specific laboratory profile [7, 50, 51]. It should be emphasized that circulating lymphocytes represent only 2% of lymphocyte population; the vast majority of lymphocytes can be found in immunologically active tissues, such as lymph nodes, spleen, intestines, blood marrow, thymus and skin [52]. During heavy training, some tissue lymphocytes may migrate to the circulation in response to hormonal stimulation typical for strenuous exercise (e.g. catecholamines, cortisol). During post-exercise recovery, these lymphocytes migrate back to tissues, especially to those generating strong inflammatory/chemotactic signals [53]. This hypothesis seems to be supported by the post-exercise increase in Treg count and subsequent post-recovery normalization of this parameter, observed during the 2nd examination in the placebo group (Fig. 1a).

ANOVA did not demonstrate a significant main effect of supplementation on Treg count; nevertheless, athletes from the supplemented group did not show a significant post-exercise increase in this parameter during the 2nd examination (Fig. 1a). Therefore, it can be assumed that supplementation with SPR played a role in the maintenance of lower Treg counts in tissues, preventing immunosuppressive effect of these cells and restoring an immune balance.

During the 2nd examination, athletes from the supplemented group presented with significantly lower pre-exercise and post-exercise values of Treg/CTL ratio compared to subjects from the placebo group (Fig. 2d). Lower values of Treg/CTL ratio in the supplemented group might also reflect a beneficial effect of SPR supplementation. The decrease in this parameter implies that SPR might mitigate the inhibitory effect of Tregs on CTLs. Due to lesser immune deficit, athletes from the supplemented group might have been better protected against opportunistic infections and reactivation of latent viral infections (e.g., with CMV, EBV and HSV-1). In turn, higher values of Treg/CTL ratio in the placebo group might reflect an unfavorable shift in the “overtraining threshold” associated with a radical deterioration of immunity.

Irrespective of the examination term and the study group, we observed a significant post-recovery increase in Treg/Tδγ ratio (Fig. 2c). This effect was modulated solely by exercise. Nevertheless, during the 2nd examination we observed a significant post-recovery decrease in Tδγ cell count in the placebo group, but not in the supplemented group (Fig. 1c). Due to presumable protective effect of SPR, preventing the post-recovery decrease in Tδγ cell count, strenuous exercise had probably less detrimental effect on the immune function in supplemented athletes. Tδγ cells play a key role in antibacterial, antiviral and antitumor immunity. Published evidence suggests that the number of Tδγ cells is inter alia modulated by exercise intensity. Anane et al. [1] demonstrated that either high- or low-intensity exercise stimulated an increase in the number of circulating Tδγ cells in previously untrained persons. However, another study showed that escalation of training loads during a winter training season resulted in a decrease in Tδγ cell counts in peripheral blood of elite swimmers [54].

Published data imply that SPR may exert a substantial effect on the activation of NK cells and their cytotoxic potential [35, 37, 43]. We did not observe a significant main effect of supplementation on either NK cell count (Fig. 1b) or Treg/NK ratio (Fig. 2b); however, irrespective of the examination term, strenuous exercise stimulated a significant increase in the number of NK cells (Fig. 1b).

According to a widely accepted concept, strenuous exercise stimulates migration of tissue lymphocytes to peripheral blood in a catecholamine-dependent mechanism. This hypothesis is inter alia supported by an increase in circulating lymphocyte count observed after infusion of epinephrine [55, 56]. However, the fact that the post-exercise increase in lymphocyte count in elite athletes was less evident than in untrained persons, implies that immune response of the former group to strenuous exercise may be weaker [57, 58].

Post-exercise changes in lymphocyte count in intensively trained athletes were markedly less evident than in untrained persons, and less pronounced than it could be expected based on epinephrine and cortisol concentrations. However, published evidence suggests that changes in hormonal parameters do not necessarily correlate with lesser mobilization of tissue lymphocytes and their migration to peripheral blood [8, 10]. Therefore, lesser responsiveness of tissue lymphocytes from elite athletes to strenuous exercise was interpreted as a consequence of training-induced changes, such as a decrease in the reactivity of ß2-adrenergic receptors on these cells, altered expression of adhesive molecules or their ligands [57, 58].

However, in our opinion, also other potential mechanisms contributing to reduced mobilization of tissue lymphocytes after strenuous exercise need to be considered. According to a widely accepted and empirically verified hypothesis, various stimuli (among them physical exercise and infections) may induce bidirectional flow of lymphocytes from/to tissues (spleen, muscles, lungs, bone marrow) [9]. Adams et al. [9] showed that exercise promotes rapid migration of tissue lymphocytes (primarily from the spleen and lungs) to the circulation, along with markedly less evident shift of these cells from muscles to blood. Although this observation originates from a study in rats, the post-exercise increase in circulating lymphocyte counts in humans may also, to a certain degree, reflect higher absolute number of these cells in the spleen and lungs (and to a lesser extent, in muscles). Less evident post-exercise migration of lymphocytes to the peripheral blood of well-trained athletes may be associated with lower number of cytotoxic cells in their spleen and lungs, and/or with higher number of tissue Tregs. The latter hypothesis is also supported by the results of our study, which demonstrated a significant post-exercise increase in Treg count in the placebo group during the 2nd examination. However, also lymphopenia, observed few hours post-exercise as a consequence of the recirculation of blood lymphocytes to tissues (only < 10% of circulating lymphocytes undergo apoptosis), is a well-established phenomenon [10]. During post-exercise recovery of well-trained athletes, their lymphocytes may primarily recirculate to the muscles; due to multiple training-induced microinjuries, muscle tissue synthesizes large volumes of cytokines which act as a chemoattractant for peripheral lymphocytes [59, 60].

According to Adams et al. [9], recirculation of lymphocytes from muscles to the blood is less pronounced and slower than between spleen or lungs and peripheral circulation. Probably, lymphocytes of well-trained athletes may remain anchored in muscles for a longer time, which makes them less prone to recirculation and more susceptible to apoptosis. This may result in permanent loss of some cytotoxic lymphocytes from the spleen and lungs. However, the exact reason behind a post-exercise increase in Treg count in well-trained athletes is still unclear. Perhaps, this phenomenon is associated with an increase in IL-2 (synthesized by T cells in response to repeated strenuous exercise), stabilization of Foxp3, longer survival of mature Tregs, and resultant increase in their number in some tissues [61, 62].

Our findings suggest that another, yet unidentified mechanism may exist behind the post-exercise immune impairment observed in elite athletes subjected to heavy training loads. Higher number of circulating Tregs may reflect their increased counts in immunologically active organs, presumably in the spleen and lungs. The number of these tissue Tregs that migrate to the circulation during strenuous exercise may be higher than the number of migratory cytotoxic T lymphocytes (other than NK cells). Furthermore, it cannot be excluded that immunologically active organs are not only abundant in Tregs, but also contain less cytotoxic lymphocytes (Tδγ, CTL). During strenuous exercise, these sparse cytotoxic cells may migrate to other tissues (primarily muscles) whereby they undergo apoptosis, which probably also contributes to a post-exercise immunity impairment.

Our present study showed, for the first time, that supplementation with SPR may modulate some components of the immune system in athletes exposed to repeated strenuous exercise. The fact that rowers from the supplemented group did not show a post-exercise increase in Treg count (Fig. 1a) implies that SPR may play a role in maintaining normal tissue level of these cells during strenuous exercise, thus preventing immunosuppression. Moreover, SPR seemed to attenuate a suppressive effect of Tregs on CTLs, since during the 2nd examination, athletes from the supplemented group presented with significantly lower pre-exercise and post-exercise values of Treg/CTL ratio than subjects from the placebo group. Finally, strenuous physical exercise did not exert a significant effect on Tδγ cell count in the supplemented group, whereas athletes from the placebo group showed a post-recovery decrease in this parameter. Altogether, these findings suggest that supplementation with SPR may exert a beneficial effect on selected components of the immune system in athletes exposed to heavy training loads.

Future studies should center around better understanding of the mechanisms of immune impairment activated during and after strenuous exercise. Another direction of future research should be the identification of factors that may counterbalance the unfavorable consequences of exposure to maximal training loads.

## Conclusions

The results of this study imply that supplementation with SPR extract might prevent a post-exercise increase in Treg counts and a post-recovery decrease in the number of Tδγ cells, which contributed to lower Treg/CTL pre-exercise and post-exercise values ratio during the 2nd examination. Altogether, these findings suggest that supplementation with SPR extract may protect athletes against a deficit in immune function (especially, anti-infectious function) associated with strenuous exercise, and may cause a beneficial shift in an “overtraining threshold” that prevents a radical deterioration of immunity.

## Abbreviations

CD: Cluster of differentiation
CMV: Cytomegalovirus
CTL: Cytotoxic T lymphocyte
DCs: Dendritic cells
EBV: Epstein-Barr virus
FOXP3: Forkhead box P3
HIV: Human immunodeficiency virus
HSV: Herpes simplex viruses
IL: Interleukin
INT-γ: Interferon gamma
LA: Lactic acid
NK: Natural killer
RDA: Recommended dietary allowance
SPR: Spirulina platensis
TNF alpha: Tumor necrosis factor alpha
Treg: Regulatory T cells
Tδγ: T cells gamma delta
